# Psychiatry as Mind-shaping

**DOI:** 10.1007/s10670-024-00908-2

**Published:** 2024-12-16

**Authors:** Jodie Louise Russell

**Affiliations:** Institute for Mental Health, School of Psychology, College of Life and Environmental Sciences, University of Birmingham, Birmingham, UK

## Abstract

I argue that psychiatric researchers, clinicians, and the wider public actively regulate the minds of individuals with mental disorder through the prescriptive processes of mind-shaping (see Andrews in South J Philos 53:50–67, 2015a; Andrews in Philos Explor 18(2):282–296, 2015b; McGeer, in: Folk psychology re-assessed, Springer, Berlin, 2007; McGeer in Philos Explor 18:259–281, 2015; Mameli in Biol Philos 16(5):595–626, 2001; Zawidzki in Philos Explor 11(3):193–210, 2008; Zawidzki, in: Kiverstein (ed) The Routledge Handbook of Philosophy of the Social Mind, Taylor and Francis Group, London, 2016). Consequently, all those with a vested interest in the language of mental disorder should take a critical and dialogical approach in how concepts of psychopathology are developed, disseminated and used. Mind-shaping describes how our folk-psychological categorizations actively regulate the behaviour of those categorized. This is done through setting certain norms, which can be achieved through the application of folk-psychological concepts. I argue that psychiatry has embedded norms and goals in its activities that are non-epistemic in nature and these are not only bound up in disorder concepts, but also in the social roles that clinicians, researchers and patients play. In this way, psychiatry uses folk-psychological type tools for the social understanding of individuals with mental disorder, and the application of these tools also helps it meet these non-epistemic goals. Given this, I characterise psychiatry as partaking in mind-shaping. When we characterise psychiatry as mind-shaping, we are then able to explain occurrences of looping effects between disorder categories and individuals categorized (Haslam in J Psychopathol 22(1):4–9, 2016) and provide a theoretical basis for the occurrence of hermeneutical injustice in the field of mental health.

## Introduction

In this paper, I argue that clinicians and researchers in psychiatry participate in processes of ‘mind-shaping’—a family of approaches that characterise social cognition as the regulation and shaping of another’s mind (see Andrews, 2015a, 2015b; McGeer, 2007, 2015; Mameli, 2001; Zawidzki, 2008, 2016). One set of tools that individuals use to ‘shape’ the cognition of others are folk-psychological concepts, which are normatively laden categories that are applied to people to guide their behaviour. I demonstrate that psychiatry makes similar use of concepts to guide behaviour, either intentionally or unintentionally, for instance, through the use of disorder concepts. I argue further that these mind-shaping effects can have potentially harmful consequences for individuals with mental disorder that have been hitherto overlooked. This should lead clinicians, researchers and the wider public, ultimately, to take a critical and dialogical stance toward psychiatric research, and to consider further whether it ‘shapes’ minds for the better.

The idea that disorder concepts in particular shape the behaviour of whom they are applied to was famously expounded by Ian Hacking in his paper “The looping effects of human kinds” (1995a). Hacking argued that individuals respond to being labelled, and such labelling informs how they behave which also shifts the definition of the original label to fit the new behaviour. Hacking notes looping effects in psychiatry, such as in the history of multiple personality disorder where the increased rate of diagnosis of the disorder led to a widening of its symptoms and an increase in the number of alters an individual was expected to have, as ‘eliciting’ alters became part of the therapeutic practice for treating the disorder (Hacking, 1995b). It is because individuals not only react to being labelled but are also treated in particular ways because of a label (which affects the individual being labelled herself) that Hacking refers to human kinds as ‘moving targets’. I will develop and expand Hacking’s original thesis by providing a theoretical framework to describe how and why such looping effects take place. Importantly, in further development of Hacking’s work, I outline how this process can be both helpful and harmful to the individuals being categorized as well as what we, as researchers, should consider moving forward.

One way in which psychiatric practices may initially be seen to influence the targets of its understanding, i.e. people with mental disorder, is in the influence of embedded values in clinical practice and research. Recent work in the philosophy and history of psychiatry does well to point out this relationship; Bueter (2019), for instance, argues that the values regarding who counts as having ‘expertise’ relevant to taxonomic decision making in psychiatry results in hermeneutical and testimonial injustice, two forms of epistemic injustice, when patient input is excluded from this process. Hermeneutic injustice is where individuals suffer harms due to a lack of conceptual tools, or inability to develop said tools, which the individual could use to understand her own experience. Testimonial injustice is where a person’s experience is excluded from knowledge generation processes, such as when doctors are treating their patients, on the basis of some feature of the person which, from the perspective of another, rules them out as an adequate ‘knower’ (see Fricker, 2007). Harm is understood here to be a kind of dehumanisation as a result of one’s knowledge as a person being degraded; one is treated as having less than full capacity as a knower, and thus is less of a person (as in testimonial injustice), or someone’s capacity as a knower is genuinely compromised which does in some important way prevent someone participating fully in the activities of persons (as in hermeneutical injustice). Although it has been argued that the existence of epistemic injustice itself doesn’t beget a harm (see Dunne & Kotsonis, 2024), for my purposes it is sufficient that epistemic injustices risk these kinds of harms, and this warrants a critical discussion of the shaping effects of values in psychiatry.

However, the existence of epistemic injustice does not itself explain the looping effects that Hacking describes. More needs to be done in philosophy of psychiatry to understand the mechanisms behind psychiatric values, namely, how they are able to shape the behaviour of individuals that result in harms such as epistemic injustice. I intend to fill this gap through the mind-shaping view to account for how psychiatric practices come to have a prescriptive force that shapes behaviour and experience.

It should be noted that the connection between the mind-shaping thesis and mental disorder has already been expounded by enactivist proponents like Michelle Maiese. In *Autonomy, Enactivism and Mental Disorder* (2022), Maiese argues that the processes of mind-shaping themselves can be responsible for experiences of disorder. This is because, Maiese argues, mind-shaping can result in overly rigid social scripts and habits that prevent us from flexibly adapting to or critiquing the challenges of the environment. For Maiese, the cornerstone of mental health is a reasonable amount of autonomy and agency; when mind-shaping processes are coercive to the extent that these scripts are internalised and someone’s autonomy is undermined, cases of mental disorder can arise. In contrast, I use mind-shaping instead to understand the effects that scientific research and clinical practice has on the looping effects of disorder categorization and phenomenology, rather than as a factor that contributes to the generation of pathological experience itself.

I will begin by outlining the mind-shaping view to show how our folk-psychological concepts shape and mould the behaviour of the individuals we classify (Sect. 1). Then I will demonstrate how psychiatry participates in processes of mind-shaping by using disorder categories much like folk-psychological categories, and by setting ‘norms’ for psychiatric practice and research that guide behaviour (Sect. 2). Lastly, I spell out the consequences of this view in terms of the harms and benefits that mind-shaping may cause to those with mental disorder (Sect. 3) before briefly concluding this discussion with considerations on concessions we must make when trying to understand pathological experiences (Sect. 4).

## Mind-shaping

Traditional, or orthodox, accounts of social cognition adopt a “theory of mind” model to social understanding, where individuals are hypothesised to be ascribing mental states to others, correctly or incorrectly. This may be done through, for instance, an internal model of another’s mind which carries out a simulation of their mental states (as in Simulation Theory accounts) or through some folk-psychological theory of what the other is thinking (as in Theory Theory accounts) (Gopnik & Wellman, 1992). For example, I may ‘read’ the mind of my partner, who is wistfully looking toward the kitchen, by simulating their mental state using my own internal model of their mind. Given the accuracy of this model, I successfully attribute to them, based on this model, the mental state of hunger. Alternatively, on the Theory Theory account, I may have a folk-psychological theory that when people tend to look forlorn and glance towards the kitchen, they are typically in the state of ‘hunger’, and this is how I am able to successfully attribute the mental state of hunger to my partner. In this way, orthodox accounts understand social cognition to be more descriptive or predictive in nature. Mind-shaping (Mameli, 2001; McGeer, 2007, 2015; Zawidzki, 2008, 2013, 2016), in contrast, characterises social cognition as more prescriptive and regulative; it is the process of understanding each other through conforming to sets of shared norms. These norms are derived from folk-psychological classification. For example, instead of attributing the mental state of ‘hunger’ to my partner, I instead read their behaviour as conforming to the norms of the folk-psychological category ‘hungry person’; a hungry person acts in particular ways (like looking wistfully at the kitchen), which my partner may conform to—intentionally or unintentionally—to communicate to me that they are hungry.

This regulative dynamic, I argue in the next section, makes a difference in the case of the scientific pursuit to understand human experience once we understand that scientific practice is likewise prescriptive and normative, even when it is trying to uncover ‘natural’ features of the world.

Scientific practices can take on normative significance because the agents involved, i.e., in the case of psychiatry, researchers, clinicians and patients, are ultimately social agents, and social agents have a stake in being intelligible to others (McGeer, 2007). Our survival as organisms appears to hinge highly on navigating the environment successfully, which includes other social organisms. It’s important, therefore, to be able to understand another’s intentions, and it behoves us to do our best to try and make ourselves understandable to others; how we are understood by others might also impact our chances for survival. It’s useful, for instance, to have an agreed upon understanding of ‘predator’ and ‘food’ as well as a shared schema for what to do in instances that involve these things (e.g. fight or flee). Understandability is something always at stake in social coordination tasks and coordination over time is not only beneficial but essential; for us to work on environmental tasks together, especially as I am likely to have to work with many of the same people more than once, coordination gets off the ground quicker and more easily if individuals have a pre-established history that they can draw on.

In order to achieve a foundation of understandability for coordination, we conform, or encourage others to conform, to a set of norms under the mind-shaping account. These norms come from folk-psychological categories, our psychological tool set for classifying people, which is what we use to ‘make sense’ of another’s behaviour. For example, if someone were to walk into my classroom, they can easily identify me as the ‘teacher’ without having met me before because I may conform to certain teacher norms (e.g. I stand in front of the class, I speak with a certain level of authority, I take the register etc.) On this basis, someone may then know how to act appropriately in this space (e.g. to take a seat at a desk). Folk-psychological categories don’t just describe what someone is like or how they behave but encourage people to act in accordance with the norms of that category. For example, in treating someone as a teacher, based on our categorization of them as a teacher, we shape that individual to act in ‘teacher-like’ ways, either intentionally or unintentionally. Our behaviour can also reveal the norms we are conforming to, but this also comes with expectations about how one should behave, given these norms (Mameli, 2001). If a person ‘performs’ as a teacher (i.e., acts like one), or are categorized as such, then they should, for instance, set homework and do other typical teacher activities. It is these expectations that are built into the folk-psychological tools we use to understand people that makes the processes of mind-shaping prescriptive and regulative in nature. Moreover, since we are likely to meet the same individuals repeatedly, it is beneficial to maintain the same norms on repeat interactions, which generates what Mameli (2001) terms ‘the expectancy effect’. This is where past expectations regulate behaviour in the future in service of continued social coordination. Folk-psychological categories are thus prescriptive over time too.

In addition to this, by acting in ways that conform to particular norms of the folk-psychological category applied to me, and the expectations this comes with, I put those norms ‘on the table’ for other people to respond to in other norm-conforming ways (e.g. the existence of teacher-types in the room may encourage me to act in student-type ways). Thus folk-psychological categories under mind-shaping draw close parallels to Hacking’s (1995a) ‘human kinds’ and the looping effects they exhibit. Particular to the ‘human kinds’ under mind shaping, however, is that folk-psychological categories shape behaviour through setting expectations. Mind-shaping thus provides a convincing account of how Hacking’s human kinds have the prescriptive force that they do. In addition to this, norms can determine the available or ‘playable’ moves in social interactions, much like the rules of a game like chess determine what counts as a proper chess move (or even define the nature of the game itself) (McGeer, 2015), which helps to explain why folk-psychological categories shape us in particular ways; the norms themselves constrict possible ways of interacting in the social situation itself. If I am categorized as a teacher, picking up ‘student’ norms is prescriptively discouraged as a possibility to me (I risk alienating and confusing others in the room by acting in unexpected ways which could be detrimental for coordinating on problems in the environment), although I have the option of being a ‘strict’ teacher or a ‘fair’ one. To relate this back to the case of mental disorder, expectations set by researchers, mental health practitioners, the wider public, and individuals themselves, will be bound up in the concepts applied if these concepts exhibit similar features to folk-psychological categories. I argue in Sect. 2 that this is, indeed, the case. This means further that concepts applied to individuals with mental disorder, such as disorder concepts, diagnostic categories, or even concepts like ‘patient’, might restrict what those individuals are able to do in a space, given the expectations placed on them about how they should behave.

In addition to the above, I claim that the normative practices that arise from social interaction don’t just constrain what may be possible to understand or ‘perform’ in a social space but they can also constrain our interaction with the environment at large. Expectations surrounding the folk-psychological concept of ‘woman’, for instance, places constraints not just on how someone interacts with people of other genders but the expectations that come with that folk-psychological concept may constrain how they inhabit and interact with the physical space (how they can sit, how they can throw a ball, etc.; Mameli, 2001; Young, 1980). To put it another way, folk psychological categories can change the structure and bounds of the ‘I can’ in our experience (Merleau-Ponty, 2014). This stronger claim is warranted in order to make sense of how looping effects don’t just pertain to behaviour in the social realm; our concept of teacher might change in terms of their behaviour towards others (from a strict disciplinarian to a nurturing and encouraging presence), and this then informs what it means to be “the teacher”, but the physical tools and space for the teacher (the chalk, the blackboard, the classroom) will also constrain and inform their role. Teachers now, for instance, have to reconcile their goals with the presence of smartphones in the classroom; this itself can be a bane to the teacher or an educational tool, depending on what they decide to do, and what they decide to do will then inform the kind of teacher that they are (and what it means to be a teacher at large).

Furthermore, by applying the term ‘teacher’ to an individual, we may also experience the environment as a particular space for particular (norm-guided) activities; the term ‘teacher’ is closely associated with the term ‘classroom’, and thus by labelling someone as the teacher, one might also determine that the room in which they occupy must be used for teaching activities (and thus is appropriately labelled the ‘classroom’). One should not, for example, have meetings in the classroom (at least, not when class is being held), make lunch in the classroom, or try and buy books in the classroom. Instead, one might be expected to participate in ‘learning’ activities—whatever these might entail—given the classification of ‘teacher’ and ‘classroom’. In this way, what one can do in a given space may importantly be determined not only by the folk-psychological categories we apply to people in the space, but also the implications of these categories for the use of the space.

This is relevant in the case of mental disorder where what one feels one can or cannot do may be constrained by mind-shaping processes, and this may shape wider research and clinical understandings of what it means to be a person with mental disorder when clinical and scientific knowledge is informed by the behaviour of those with mental disorder. For instance, the participants in Örmon et al.’s (2014) study of experience of care from women that are victims of abuse noted that clinicians were fixated on the process of diagnosing mental disorder rather than validating the women’s experiences of abuse. As such, to be taken seriously, the participants noted that, in particular, “a bipolar disorder diagnosis was automatically ‘worthy of care’ because of the serious nature of the disease and were thus listened to” (Örmon et al., 2014, p. 2307). This led to women conforming to certain scripts or “behavioural codes” in order to conform to disorder-type categories so that they would be taken more seriously. They describe, for example “dressing in hospital clothes, not wearing make-up and crying” (Örmon et al., 2014, p. 2307) in order to receive care. In this way, the women in Örmon et al.’s study conformed to norms and expectations around what it means to be a person with mental disorder in order to be understood under the label of ‘mentally unwell’, and receive treatment they might have otherwise been denied, as a result of this labelling.

That is not to say that these women were pretending to be mentally ill to receive care; these women conformed to certain norms of behaviour for mentally ill people that would be specifically understood by clinical staff they interacted with as mental illness. The experiences of abuse that these women disclosed, however, were not always considered to be relevant to the diagnosis of mental illness (Örmon et al., 2014), hence these women may have felt it necessary to act in stereotyped or typical ways. In the clinical environment, we might hypothesise that the only way to get care at all is to conform to particular mental illness norms, which may themselves not be accurately indicative of mental illness. With very few options for receiving appropriate care, we might say that these women were mind-shaped into behaving in disorder-like ways as dictated by psychiatric norms of what mental disorder behaviour looks like. The consequence of this, however, was that the women’s struggle to have their abuse recognised and their responsibility absolved was often attributed to mental illness itself by clinical staff, or their abuse experiences were often considered secondary to their mental illness. In this way, the women’s struggles either confirmed the mental illness diagnosis or were dismissed to reaffirm the norms of the diagnosis. As Findlay (in Lee, 2013) notes in relation to interacting with clinicians:

The shrinks had a corner on my reality… Because I was mentally ill I had no credibility: they could believe my answers or not, as they chose. If my answers were wrong, I was denying the problem; if I disagreed with them I was hostile and/or experiencing resistance to treatment… If I refused to answer their questions I was resistant; if I told them it didn’t matter I was denying. There was no way to convince them otherwise… They got to decide what was true and real for me. (Lee, 2013, pp. 62–63)

Findlay and the participants of Örmon et al.’s study demonstrate that conforming to mental illness norms can be a catch-22; if you conform to them, you are deemed mentally unwell, but, perhaps, in a way that doesn’t get at the problem, and if you resist them, you are deemed mentally ill from the resistance itself. However, for our purposes, the examples above serve to demonstrate not only how mind-shaping can occur to produce self-reinforcing looping effects through setting norms and expectations, it also demonstrates that there are plausible cases of mind-shaping in the psychiatric domain. However, more must be said here to make a strong case for how psychiatry, on a more institutional level, participates in mind-shaping processes. It is also implicit in the examples above that clinical practitioners in particular have a lot of power in setting norms during mind-shaping, but it is not yet explicitly evident how this has come about. This is what I turn to in the next section.

## Psychiatry as Mind-shaping

Initially, scientific human kinds, like those in psychiatry, may not seem to share the same prescriptive and normative aspects as folk-psychological categories, like ‘teacher’, from a mind-shaping perspective. We might think that the scientific study of mental disorder is meaningfully separated from every day folk-psychological use of disorder terms because science doesn’t come with prescriptions, values or norms; scientific terms and concepts may reflect or refer to natural (or even social) facts. In the case of the women with trauma in Örmon et al’s (2014) study above, the women were responding to the added social consequences of having a mental disorder (i.e. that it makes you eligible for care), but one might argue that this is separate and incidental to the natural fact of having a disorder, which is what psychiatry is concerned with. Based on this, one might therefore argue that psychiatry doesn’t try to influence or mould individuals into normative frameworks in order to understand them, but mostly categorises so as to learn something about people (not for social ends like social coordination via mind-shaping). In this way, scientific concepts like ‘disorder’ and ‘dysfunction’ might not come under the purview of mind-shaping, and thus the epistemic harms I outline in Sect. 3 are irrelevant.

In order to motivate the claim that psychiatry—both research and clinical practice—participates in mind-shaping, I must first show that the medical sciences are not solely in the business of making descriptive, naturalistic claims. Instead, I must show that psychiatry, either intentionally or unintentionally, makes prescriptive claims. To do so, I will first show that psychiatric claims are norm and value laden, drawing on the notions of” epistemic” and “non-epistemic” values in science. By showing that psychiatry has embedded norms, values, and interests, I will then provide in principle support for the claim that work in the domain of psychiatry constructs and shapes individuals along these values and norms.

Epistemic values, broadly construed, refer to those kinds proposed by Thomas Kuhn (1962) which are meant to guide scientists in theory choice in line with knowledge and truth-seeking goals, and may include, for instance, the values of fruitfulness or accuracy (Rooney, 1992). Non-epistemic values include social, cultural and personal values and may similarly inform scientific theory choice, intentionally or unintentionally, such as consideration of a theory’s contribution to human flourishing (Kourany, 2003; Longino, 1995; Rooney, 1992).^1^ Feminist philosophers of science argue that scientific research is influenced by both epistemic and non-epistemic values; for instance, the scientific pursuit of particular facts is shaped by human interest in those facts (for example, by the pursuit of some facts over others, and by the idea that there may be facts to discover at all). In what follows, I will show that psychiatry as a science has embedded non-epistemic values which generate non-incidental social consequences (Sect. 2.1). These social norms and values embedded in psychiatry also shape the individuals it studies and treats as a result of those values and norms (Sect. 2.2). We can characterise this impact as mind-shaping because, as I argue in Sect. 2.3, the scientific interest of psychiatry is to understand people and thus its activity is a kind of social cognition.

### Psychiatry as a Value-laden Science

Evidence of the involvement of blatant non-epistemic values can be found, for example, in the criticisms levied against the developers of the Diagnostic and Statistical Manual of Mental Disorders (DSM), for their close ties to the pharmaceutical industry (Cooper, 2017), which has clear financial stakes in who gets classified as disordered for selling medication.

More surreptitiously, however, we may see values to have crept in to particular diagnostic categories such as in the case of personality disorders. Watts (2019) claims, for instance, that the criteria for personality disorders in the 11th edition of the International Classification of Disease (ICD-11) were purposefully widened in order to capture the much wider prevalence of the disorder than is currently reflected in the number of people being diagnosed. As a consequence, the number of patients diagnosed with personality disorders increased with the use of the ICD-11 (Watts, 2019). One may argue here that the widening of the criteria is for purely epistemic reasons; the World Health Organisation arguably adjusted the criteria in order to better capture the proportion of cases of personality disorder in nature, and thus the decision has more to do with reflecting some knowledge or truth about the world than other human interests. However, we have reason to doubt that such decision-making, when it comes to psychiatric classification and practice, are the result of exclusively epistemic values. Wallace (2019) notes, for instance, that the definition of mental disorder was broadened in the UK’s Mental Health Act in order to include personality disorders so that those detained under the act would have access to appropriate care. One might argue that there are epistemic values in play here (i.e. by broadening what we think ‘mental disorder’ refers to in the world) but the goal of improving access to care seems non-epistemic in nature; access to care, after all, doesn’t tell us itself about the legitimacy of the label of ‘mental disorder’ within a scientific context, but reflects, instead, our social values that people under certain labels should have access to particular resources.

Wallace (2019) highlights, however, that the principles behind personality disorder diagnosis are meant to be neutral and objective, where the social factors which contextualise one’s behaviour are meant to be excluded from the assessment. In reality, Wallace argues further, diagnosis involves making subjective judgements about appropriateness of these behaviours, and certain contextual factors (like the patient’s gender) can skew the clinician’s judgment. For instance, the diagnostic criteria for Borderline Personality Disorder (BPD) in the fifth edition of the DSM (DSM-V) includes the symptom “emotional instability in reaction to day-to-day events, such as episodic sadness, irritability, or anxiety”. Women are often stereotyped to be overly emotional, compared to men, and this has been gestured to as one of the possible reasons that BPD is diagnosed more frequently in women than in men (Bjorklund, 2006). It is also worth noting that, even though it may be argued to be a useful diagnosis and has brought much understanding to patients and clinicians alike, there is little to no empirical research to suggest it is a unified disorder in and of itself (Tyrer et al., 2019). This suggests that it is not the case that purely epistemic values—in the pursuit of knowledge or truth—were involved in the construction of BPD as a distinct disorder category and some of its criteria may reflect societal biases or goals more than a distinct ‘joint’ in nature.

As a result of embedded values in diagnostic criteria, Watts (2019) notes that there are additional “shaping effects” (p. 462) in the application of the diagnostic label of personality disorder on clinical attitudes to patients, describing that “the idea of a personality disorder often prejudices clinicians to situate symptoms of distress as manipulative, attention-seeking, and wilful” (p. 462). Due to negative stigma towards individuals with personality disorder, the behaviours of patients are often reinterpreted under the lens of the personality disorder label. These perceived symptoms may then be delegitimised and given less credibility in order to focus on what the clinicians judge to be the ‘main’ disorder at play (Örmon et al., 2014). The downplaying of personality disorder symptoms, then, feasibly feeds into the perception of those with personality disorders as timewasters or attention seekers. Wallace (2019) similarly notes that individuals with BPD are often perceived as ‘threats’ to a clinician’s professional self, which is mitigated by the clinicians through distancing strategies. This involves, for example, referring to clients by their diagnosis in order to deindividualize them. As a result, Wallace notes, clients’ perspectives were often doubted, and clients would also be referred elsewhere, only to increase the distress of the client. This sometimes led to the client reapproaching the mental health services in more destress, which perpetuated a cycle of increasing distress and mistrust (Wallace, 2019).

Non-epistemic values are therefore in play in the development of scientific concepts in psychiatry, as well as in clinical treatment, and these all have tangible effects on those that psychiatry studies; the case of personality disorder above shows that how the category is constructed determines whether someone gets treated, or not, and even the quality of treatment they might receive. This can affect patient behaviour in turn. Thus, the case study of BPD demonstrates, furthermore, that medical concepts constrain possibilities for action through setting expectations as to what it is to ‘be like’ a person with this diagnosis. A person with BPD, according to the diagnostic criteria and the stigma associated with the label, is emotionally unstable. However, the inability to regulate emotional experience may be exacerbated by the fact that the label itself puts patients ‘at arm’s length’, with little agency over their own care. Just as individuals with BPD understand their behaviour through the label of ‘BPD’, which is front-loaded with particular norms as to how individuals with BPD act and experience the world, other disorder concepts are also likely to be bound up with norms and expectations. There is a specific way *to be* for someone with depression, anxiety or BPD, for example, which is defined by the norms associated with these concepts that set expectations about how one with any of these disorders should behave and experience the world. The expectations built into specific psychiatric concepts, then, based on the examples above, exhibit the behaviour-regulating effects of folk-psychological categories according to the mind-shaping hypothesis.

### Psychiatry as Shaping the Clinical Domain

Additional support for the influence of non-epistemic, and social, goals in psychiatry, lending weight to the argument it partakes in mind-shaping, can be found in the history of medicine. Sadler (1978), for example, characterises the history of medical institutions in the UK as a struggle for power between different ideologies with different conceptions of what medicine is (medicine as art and medicine as science) and this led to medicine being practiced in different ways before it was standardised. For Sadler, conceptualising medicine as a science involves norms and values of determinateness; a scientific approach is one which attempts to box, standardise and technologize its subject. Conceptualising medicine as an art, in contrast, characterises medicine as indeterminate, with an emphasis on experience and tailoring treatment to the individual. Sadler’s argument implies that what one wants to call ‘medicine’ in the first place, is down to the specific norms and practices of the people involved (rather than the facts themselves) and one need not adopt the scientific perspective on the ‘natural facts’. Sadler (1978) argues further that medicine became increasingly characterised as a science due to the way that the medical knowledge and educational institutions themselves were standardised, consolidated, specialised, and made determinate and technical in order to prohibit deviation (and thus allow full control by those with the requisite knowledge and qualifications over practicing medicine, creating medical elites). Chapman (2023) similarly makes the case that modern psychiatry arose, in part, due to pressures to legitimise itself against the critique from Thomas Szasz that mental illnesses are simply ‘problems in living’. To this end, psychiatry, as Chapman (2023) describes, attempted to standardize the ‘normal’ or typical mind according to scientific theory, deriving an idea of function from the principle of natural selection, from which mental illness was conceptualised as the ‘abnormal’ or ‘dysfunctional’ mind. Through this lens, we can say that the development of modern psychiatry came about due to adopting scientific practices and norms for reasons based on values (i.e. that scientific norms are the only ones that produce legitimate knowledge) rather than from a pure concern for the ‘natural facts’.

The way that psychiatry has historically come to be characterised and understood, i.e. as scientific, has subsequently guided and constrained what is possible or not possible for individuals to do within psychiatry; for example, if you don’t have the requisite education, certified by legitimized institutions, then you simply cannot practice or research psychiatry at all. In this way, psychiatry, as a domain of social activity, was shaped in this historical power struggle into a more scientific field, and as such psychiatry itself shapes clinicians and researchers into the appropriate kinds of people to carry out medical research and treatment in the adoption of specifically ‘scientific’ norms. In other words, by defining itself, psychiatry seems to participate in social-coordination tasks; individuals in psychiatry work together, intentionally and unintentionally, to create norms which determine what constitutes the social institutions of medicine and psychiatry (to stop, for example, ‘improper’ or ‘inappropriate’ individuals from claiming membership to these groups).

Moreover, the social-coordination of establishing psychiatry as a field justifies its own existence through the construction of the patient: the target of psychiatric understanding. As Herzlich and Pierret (1985) describe: “The conceptions that a society forms about sick people and that the sick internalize and sustain-orient, organize and legitimate social relationships and, to a certain degree, ‘produce’ the ‘reality’ of sick people” (p. 146). In the current day, Herzlich and Pierret note that patients are conceptualised as someone to be ‘cared for’ and have a prominent relationship with medical institutions. This is in contrast to the seventeenth century and earlier where sickness itself was conceptualised in Christian countries as ill fortune but also as a reflection of one’s moral character. The authors argue, therefore, that conceptions of sickness and ‘the sick’ have changed radically through time in response to changes in wider societal structure. In particular, Herzlich and Pierret argue that modern medicine came about as a result of providing a genuine alternative to religious conceptions and practices of care from the past through scientific advancement, and due to a cultural shift in sickness being a ‘way of life’ rather than a way of dying (i.e. something that impacts a person’s capacity to live or, more precisely, to work). Due to the wider cultural shift in understanding the nature of disease and illness, doctors then took over the roles previously filled by priests and nuns, and as a consequence “[t]o be ill meant calling for a doctor’s help” (Herzlich & Pierret, 1985, p. 150, emphasis added).

This coheres with Klerman’s (1977) analysis of ‘the sick role’; he argues that the medical model, through which mental disorder is primarily understood in contemporary Western psychiatry and by which disorder categories are developed, represents the patient as someone who is ‘sick’. The sick patient is implied to be, according to Klerman, both exempt from social responsibility and blame because of their disease, but they are also expected to comply with medical treatment. Medicine, therefore, in its adoption of the medical model, shapes the norms of ‘being a patient’ by constructing its target of understanding as one where medical intervention should apply. The same applies in the case of psychiatry, as an extension of medical practice and research.

Langer and Abelson (1974) note the effects of applying the term ‘patient’ on the perception of someone’s behaviour. In an experiment, two groups of clinicians were shown the same footage of an interview, but the interviewee was labelled differently for each group; for one, the interviewee was labelled as a ‘job applicant’, and for the other they were labelled as a ‘patient’. Langer and Abelson note that the clinicians in the group where the interviewee was referred to as a ‘patient’ described the interviewee as significantly more disturbed than the ‘job applicant’ group. Langer and Abelson call this a “labelling bias” and suggest that it may arise as a result of a need to filter information rich cues from other people and the environment. The authors also suggest that by simply walking into the clinical ‘space’ (be that the office of a therapist, the experiment room of a researcher), the individual inadvertently labels herself as the ‘patient’. This suggests that clinicians have certain preconceptions as to what a patient should ‘look like’ (i.e. where to find them, what kind of behaviour they exhibit, whether they are treatable) and individuals may be intentionally or unintentionally fitting this role simply by being in the clinical ‘space’. Therefore, given that psychiatry constructs itself with respect to a ‘patient’ role (in other words, psychiatric practices are orientated around the patient, without which there would be no psychiatric practice), and the significant influence psychiatry seems to have in determining that role,^2^ psychiatry can be said to be shaping its target of interest (patients) according to its epistemic and non-epistemic values.

### Rebuttals

In spite of my argument above, one might protest that psychiatric kinds are not folk-psychological because, while members of the public and many researchers and clinicians might apply these concepts to people, psychiatric kinds actually pertain to norms of functioning, and therefore they are biological, rather than social, norms. Psychiatric kinds also make reference to ‘parts’, e.g. a person’s brain states, genetics, or hormones as in the ‘chemical imbalance’ theory, in relation to disorder concepts, which means, critics may argue, that disorder concepts are separable from the person as a social organism. Psychiatric concepts, from this perspective, refer to facts in nature, not types of ‘people’ as such. Even Wakefield’s (1992) more moderate ‘harmful dysfunction’ concept, although it incorporates a more socially informed concept of ‘harm’, might be such an instance as he defines dysfunction in terms of the evolutionarily selected effects of a mental mechanism. Critics might suggest overall, then, that particular concepts of mental disorder are natural kinds and they don’t differentiate types of people at all, regardless of how the ‘folk’ may use such terms.

However, regardless of what underpins the concept you are claiming to uncover, whether it is a part of the human body or dysfunctions within the environment, the very point of having a concept of mental disorder is to differentiate pathological experiences and behaviours, as they pertain to an individual’s life, from the non-pathological. For example, the ‘joint’ in nature that a naturalist concept seemingly picks out is merely in service of explaining the differentiation between disordered and non-disordered persons. The ‘person’ afflicted with a particular disorder is always an important and relevant context for whatever disorder experience we are trying to conceptualise and define, so in beginning to investigate the nature of mental disorders, a researcher must start with the implicit assumption that some people fall into one category and others don’t. So to say that concepts of mental disorder might not pick out groups of people or ‘human kinds’ is misleading in terms of what the classificatory project is trying to achieve, which is essentially an explanation for seeming differences in people. From this perspective, concepts of mental disorder are certainly doing the job of folk-psychological categories, that is, to pick out kinds of people to understand them.

Moreover, concepts of mental disorder further imply that something should or could be done about individuals who fall under this category, in addition to providing norms for who falls under that category. This draws closer parallels to Hacking’s (1995a) prototypical case of a human kind, ‘teenage pregnancy’; having such a concept implies expectations about what individuals who fall under this category will and should do, as well as expectations about how they should be treated by others. For instance, it suggests the involvement of social services, or access to particular support structures (e.g. child care or child benefits), or even calls for wider action to be taken to prevent people falling into a particular category in the future (such as better sex education or access to contraceptives). Many of these features mirror application of mental disorder concepts, such as the implication of support structures and future prevention. This implication would be true even if no one followed through with the normatively guided actions; concepts of mental disorder have implications about treatment and intervention even if governments and institutions may fail to take the necessary steps because the concept of disorder implicitly includes the value statement that it is ‘a bad thing to have’ (Cooper 2002) and, consequently, disorder is something one ought not to have. The idea that psychiatric conceptual tools should have utility is also exemplified in the case of personality disorders. Tyrer et al. (2019) describe that a novel proposal for classification by the Work Group for Personality and Personality Disorders for the DSM-V was initially rejected by the American Psychiatric Association partially on the basis that it “was too complex to have clinical utility” (p. 482). It may be argued that utility is not the primary goal of psychiatry, either in research or clinical practice, but it nevertheless plays an important role in psychiatric decisions.

We don’t, therefore, research mental disorders for purely academic and taxonomical purposes. We may create categories to learn something about people so we know what to do about them. This is clear in the mind-shaping thesis as we use folk-psychological categories in order to grasp what norms the other person subscribes to and thus narrow the scope of appropriate action, in service of coordination. In the same way that the folk-psychological tools we use in everyday social interactions regulate the cognitive behaviour of involved agents, psychiatric categories and roles (such as ‘clinician’ and ‘patient’) likewise regulate the behaviour and social interactions of those classified as disordered. Researchers of psychopathology, under my view, are developing concepts to understand and intervene in appropriate ways in the lives of those who live with particular experiences (i.e. experiences of mental disorder) and thus these researchers partake in mind-shaping the individuals they categorize. We established at the beginning of this section that science does not operate value-free and now we can conclude that not only do individuals in the medical sciences have vested interests in the people it categorizes, and in having the authority or capacity to categorize them, but that classificatory practices of those sciences also cognitively shape these individuals, intentionally or not, according to these institutional values and norms. This is analogous to the way that mind-shaping suggests that social cognition is not simply ‘understanding’ another in a non-interactive way, but it is about regulating the other so as to coordinate on tasks which just is social understanding.

One may additionally protest that psychiatry merely has social ‘effects’ as a result of its embedded non-epistemic values; this would paint psychiatry as passive with respect to the consequences of clinical treatment and research on the self-understanding of individuals with mental disorder, which are ultimately an unintended consequence of research and clinical work. When it comes to the epistemic harms of mind-shaping (discussed below), it is clear that, on the whole, psychiatrists don’t intend to harmfully shape the cognition of individuals with mental disorder; the looping effects may simply be, in some cases, unhappy byproducts of the natural categorization that happens in psychiatric science. However, the examples have presented here show, I argue, that psychiatric practice does involve regulative, coordinated non-incidental action; psychiatry shapes, non-accidentally, and through the coordination of various actors in the psychiatric space, the roles of psychiatrists and patients along the lines of particular values that have been adopted, which may include norms regarding who counts as an appropriate individual for psychiatric treatment and research (i.e. a patient). This gives us an in principle reason to think that psychiatry has at its foundation processes for social understanding that help regulate the social-coordination task of doing “the science”. I think we wouldn’t want to say that psychiatric work happens by accident, but, perhaps, we would want to call this ‘purposeful’ instead of intentional or conscious (which would imply awareness and responsibility—an issue I will not delve into here).

Another issue one might raise is that I have focused heavily on examples of clinical cases, and instances of diagnosis which involve clinical interactions. It may be (somewhat) easy to find mind-shaping behaviour between two or more people who are directly interacting (as social behaviour can be interpreted, responsive and constraining in ‘real time’) but research in psychiatry is slow, laborious, and takes place in a variety of different mediums (like clinical trials, and qualitative interviews that may or may not get published). Thus, research may not involve human agents interacting and mind-shaping directly. One might say that without human agents directly in play, mind-shaping isn’t taking place because you don’t necessarily have people actively, in real-time, trying to understand each other’s mind like in the example I gave in Sect. 1 where I observed the behaviour of my partner to conform to the folk-psychological category of ‘hungry person’.

I argue there are nevertheless plausible instances of mind-shaping in research, and, in any case, agents do not necessarily have to be in direct contact with one another in order to mind-shape. In terms of the latter point, humans may need to decipher a range of different ‘artefacts’ from other people to glean the norms in play. For example, I may leave a note to my partner to pick up some milk; the behaviour that I am ‘shaping’ my partner to perform is clear, even when I’m not at home. We might even be shaped by the behaviour of strangers, without knowing or meeting them, such as when we follow pre-trodden paths through grass instead of the paved roads because the path is shorter or more scenic.

In terms of mind-shaping in research, Wittchen (1996) notes, for example, that there are two main theoretical approaches to comorbidity in disorder research that can greatly shape outcomes for patients: one group, referred to as “splitters”, conceptualise comorbidity as a consequence of splitting disorder categories into separate classes that actually belong together, and another, known as “lumpers”, who think more fruitful investigations may take place when we consider diagnostic categories more widely, where symptoms may span more than one disorder category. The high rate of comorbidity between anxiety and depression, for example, may be explained by splitters to be the consequence of splitting mood disorders into distinct categories, which may nevertheless, according to some splitters, be the best nosological model of these disorders. Lumpers, on the other hand, may agree that comorbidity is as a result of splitting diagnostic categories, but the more fruitful approach may be to focus on ‘mood disorders’ more broadly, rather than the finer grained categories of anxiety and depression. In this case, researchers have to make decisions regarding how comorbid cases of disorder are to be conceptualised (along the lines of ‘lumping’ or ‘splitting’) within their sample populations, or whether they are to be recruited at all. What approach one takes matters because, as Newman et al. (1998) note, the existence of one disorder may influence the efficacy of the treatment for another disorder. This could plausibly affect whether patients with comorbid disorders are then perceived as treatable, and in what way, depending on the shape and outcome of the research, which could subsequently shape how patients perceive their own comorbid disorders.

Personality disorders, for example, are highly comorbid with mood disorders (Friborg et al., 2014). Splitting these disorders into their various categories might then allow researchers and clinicians to focus on distinct treatments for each disorder. However, Perkins et al. (2018) found that diagnoses of personality disorders in particular had negative consequences for patients’ sense of identity and hope of recovery, and were associated with worries about losing access to services and poor functional value (i.e., worries that personality disorder diagnosis is not likely to lead to recovery or appropriate treatment). Indeed, Shea et al. (1992) found that patients with depression and a personality disorder were less responsive to depression treatments. One plausible reason for this is that the perspective of personality disorder as something unlikely to lead to recovery might itself be interacting with the depression treatment. If so, this would constitute a kind of mind-shaping effect where the splitting of comorbid disorders into distinct groups has led to the negative evaluation of one disorder (e.g. personality disorder) to shape the behaviour and experience of the other disorder (e.g. depression) for the individual in question. A lumping approach may plausibly result in different shaping effects; if personality disorders and mood disorders were lumped together, for instance, patients might feel more optimistic about treatment outcomes as they lack the stigmatising label of having a personality disorder. It is in this way that some of the norms of research taking place at the institutional level in psychiatry may trickle down to have shaping effects on the wider ‘folk’.

Given my answers to the problems raised above, we should conclude that psychiatry does, indeed, partake in mind-shaping processes. With this now on the table, I will now draw out the consequences of thinking about psychiatric research and treatment in this way.

## Consequences for Psychiatry Under Mind-shaping

The analysis provided so far supports Hacking’s (1995a) claim that human ‘kinds’ are “moving targets”, meaning, that the referents for human kind terms constantly shift in response to human kind labelling; the mind-shaping framework grounds this phenomenon of looping effects in processes of social understanding. This means that even for naturalistic accounts of disorder, the object of study is likely to change under the particular concept applied because of the social activities of psychiatric practice and research. Haslam (2016) notes, in evidence of looping effects in psychiatry, a trend in deepening and broadening the classifications of mental disorders in the DSM over time. Haslam notes, for example, between the DSM-I and DSM-II, new disorder categories were introduced to include sleep and eating disorders, as well as disorders specific to childhood and adolescence, which expanded the concept of mental disorder to new types of symptoms and made it applicable to new populations. Haslam warns that broader classifications in the DSM-V may create more visibility, which may also make the conceptual tools more widely available for understanding one’s condition, and thus further expand the pool of people who identify under the label further. Mind-shaping explains and predicts this trend noted by Haslam that our psychiatric concepts evolve to accommodate this shifting space of scientific research; scientists, clinicians, patients and the wider public are all actors in the mind-shaping process of trying to understand disordered experience and thus each group will respond to the normative behaviours of the others, which may mean expanding certain social roles and folk-psychological categories.

As a consequence, the expectations placed on individuals due to categorization may reinforce the category in question through “expectancy effects” (see Sect. 1) which could be problematic both for future scientific research and for the individuals being categorised themselves. In this case, a particular concept of a mental disorder might place expectations about how one should act given a particular categorization and individuals may then continue to conform to these expectations over time. Haslam (2016) argues in particular that “affected persons who hold biogenetic explanations of their own conditions tend to be more pessimistic about recovery and less confident of their capacity to exert control over their difficulties” (p. 8). With the case of depression, for instance, the idea the mental disorders are ‘inherent’ to our biological make-up in some sense may reinforce the symptoms of depression, which include low mood and lack of motivation or interest (American Psychiatric Association, 2013), by constricting possibilities of changing one’s self-conception. It is in this way that concepts of mental disorder may problematically constrain agency and self-understanding within pre-conceived conceptual boxes such that diagnosis might not actually help individuals alleviate the distress they experience. Haslanger (2019) makes a similar point in reference to disability and argues that the term ‘disability’ itself can be disabling by reinforcing the agential norms developed by people who are ‘typically’ embodied a particular way instead of challenging these norms.

For individuals who are classified as having a disorder, there is also the potential harm of both hermeneutic and testimonial injustice (see Fricker, 2007; Ritunnano, 2022). Epistemic injustice is a term that covers both testimonial injustice, whereby an individual is harmed due to perceived features of them that devalue their testimony as a knower, and hermeneutical injustice, where an individual is harmed because they lack or are prohibited access to concepts to understand their experiences (Fricker, 2007). These injustices can result in a kind of dehumanisation where individuals are either treated as less than persons due to being perceived as less credible knowers of their own experiences, or individuals are unable to partake in typical activities as persons due to a lack of hermeneutical resources. Chapman and Carel (2022) argue that, for example, due to preconceived expectations that autistic individuals suffer with autism or, even, that one must be suffering to have autism, autistic individuals are rarely seen as both happy and autistic. As such, individuals who live happily with autism have their testimonies excluded from diagnostic criteria (resulting in testimonial injustice), which thereby excludes narratives that other autistic individuals might benefit from to help understand their autism (resulting in hermeneutic injustice). In this way, autistic individuals may be dehumanised both as a result of being treated as less legitimate knowers compared to neurotypical people, and by being prohibited from contributing to knowledge around human projects, like the development of concepts of ‘the good life’. This results in a self-reinforcing looping effect where the ‘target’ of the human kind term remains, in this case, seemingly static, but only through the active, increased exclusion of contrary behaviour inconsistent with the typical features of the kind denoted by the term (in this case, autistic people that live well). Looping effects as a result of mind-shaping can therefore be harmful where the exclusion of lived experience in the feedback loop results in epistemic injustice. This occurs in the case of autism here as, by diagnosing autistic individuals with a disorder category which is associated with the expectation that these individuals suffer, only those autistic individuals who suffer will be seen to exemplify the disorder. This reinforces the idea that one must suffer if one is autistic, which many autistic individuals would dispute.

Further, by excluding some individuals from constituting a particular category entirely, scientists and clinicians who develop concepts of mental disorder run the risk of both denying individuals access to the tools which will help them understand, through the lens of norms, what their experiences mean in a medical (and also social) context. This can create harm by excluding people from avenues of medical treatment that might alleviate suffering or exclude them from participating in wider human projects like finding meaning in life or building community. It is in this way that hermeneutical injustice as a result of mind-shaping can be dehumanising. Such a cautionary tale can be seen in the case of ‘TikTok Tourette’s’ (TT) where individuals develop tic-like movements after consuming online content featuring individuals with similar tic behaviours. Müller-Vahl et al. (2022) characterise this as a mass sociogenic illness, distinct from Tourette’s Syndrome, partially due to the way that the tics are presented in the individuals afflicted. In their view, the phenomena of ‘TikTok Tourette’s’ is “the twenty-first century expression of a culture-bound stress reaction of our post-modern society emphasizing the uniqueness of individuals and valuing their alleged exceptionality, thus promoting attention-seeking behaviours and aggravating the permanent identity crisis of modern man” (p. 476). Conelea et al. (2022) emphasise exercising extreme caution in the way that we conceptualise TT, because Müller-Vahl et al.’s empirical claim that there is a significant difference between the presentation of functional tics, such as those in TT, and Tourette’s Syndrome is not well supported in the scientific literature. The authors also note that TT is disproportionately experienced by women, while traditional research on Tourette’s Syndrome has used male-dominated samples, and so there may be an implicit gender bias built into the way we investigate TT such that harmful stereotypes of women, e.g. that they are ‘hysterical’, may problematically shape the research. Conela et al. note further that much work has been done to attempt to destigmatize diagnoses like Tourette’s Syndrome and thus that language of Müller-Vahl et al. that individuals with TT may be ‘attention seeking’ greatly undermines this project. The case of TT demonstrates the problems that can arise from overlooking the mind-shaping capacities of psychiatry; not only might we characterise people with mental disorders in such a way as to be limiting to their possibilities for flourishing but we might otherwise undermine and delegitimize experiences that people find deeply distressing.^3^,^4^

These examples support my thesis that psychiatric practices and research shape the behaviour of individuals psychiatry seeks to understand and categorize, but also that the activities of psychiatry can cause harm to individuals because of their capacity to mind-shape. The framework as I have laid it out makes these potential epistemic harms more transparent so that we might be more conscious in future of how activities within the domain of psychiatry might have non-incidental, negative effects on individuals with mental disorder. An argument might be made here, however, from anti-psychiatrists that we ought to completely dismantle the harmful power structures that psychiatry can find itself imbedded in. I think that would be a mistake given that psychiatric concepts are already widely disseminated, embedded and used in social interactions in order to make sense of disordered experience. Getting rid of psychiatry would not reverse or remove the mind-shaping influence because psychiatric concepts are already deeply engrained in our wider folk-psychological toolset. Removing the individuals, spaces and tools involved would not simultaneously remove the conceptual artefacts left behind. Moreover, if we were to ban or defund psychiatric work, that also wouldn’t prohibit other individuals, disciplines or institutions fulfilling similar roles, just as medicine took over from the Church (see Sect. 2.2).

Moreover, dismantling psychiatry would undermine the good that it can do. For example, naturalistic conceptions themselves provide a means by which people can make sense and manage experiences they find distressing and thus help overcome some epistemic injustices (Degerman, 2023). The examples I have given above paint an overly negative picture of mind-shaping but I do not conclude here that mind-shaping, particularly by psychiatry, is itself harmful, only that it can do harm, especially when the various agents in the social understanding process (e.g. clinicians and service users) don’t have equal say in the trajectory of that process. I would therefore emphasise here the potential of a stronger dialogical approach to developing our concepts whereby clinicians and therapists, patients, researchers and the wider public collaborate on what our interpretive tools mean and what prescriptions are implied by them.

Tekin (2022) prescribes a similar approach. Historically, the DSM-V has excluded first-hand patient experience from informing diagnostic categories on the grounds that such accounts have not seemed, to investigators, to be epistemically rigorous (Tekin, 2022). As Tekin notes, this was due to pre-theoretical commitments to the division of “objectivity” and “subjectivity”, with objectivity being the desired quality for research in psychiatry and juxtaposed to subjectivity, which is seen to cover patients’ lived experience, a feature that cannot be detached from the data needed to ‘impartially’ inform diagnostic criteria. Tekin argues further that we should be critical of this dichotomy if we are to understand the nature of mental disorder because mental disorders are necessarily encountered and experienced by subjects. Bueter (2018, 2019) supports this, arguing that excluding patient experience not only rules out informative data for refining diagnostic criteria but this exclusion also constitutes an epistemic harm in itself where patients, as lay persons, are not considered to have valuable testimonies and, indeed, the diagnostic tools themselves may undermine patients’ credibility as witnesses to their own illness. The Participatory Interactive Objectivity (PIO) approach is developed by Tekin to address this occlusion of patient experience by reconceptualising science as a deeply social activity (albeit with epistemic goals). This mirrors my argument that we should understand psychiatry as participating in processes of mind-shaping through categorizing and conceptualising types of people for understanding, and thus doing psychiatry is itself a deeply social practice that will have its roots in wide-reaching socialised processes that underpin much of our cognitive activity. The POI approach strongly advocates for a pluralistic approach in terms of the individuals and information informing our knowledgebase in psychiatry, and understanding patients as experts in their own experiences which can usefully inform our understanding of mental disorder on a scientific level to develop better treatment (Tekin, 2022).

Cooper (2017) also emphasises, alongside user-led research, the inclusion of multiple models of mental disorder, informed by different perspectives, as a solution to the power-imbalance present in medical research (see also Tate, 2019, on taking seriously service-user narratives). This would not only better serve our empirical goals, Cooper argues, by challenging the preconceptions of academic ‘experts’, but, more importantly for my worries above, it might address overly narrow prescriptive practices in psychiatric work by providing multiple, norm-laden models to suit different kinds of people. Some individuals may prefer a bio-medical conception of disorder, for instance, but one need not be wedded to it with multiple models on the market if one finds it overly deterministic in its outlook. With this comes the potential for increased patient agency when they are offered more of a choice in how their disorder experience is understood; by offering a variety of conceptual tools that individuals are welcomed to consider and take up or reject, psychiatry itself could help combat some of the dehumanising harms of epistemic injustice by allowing the individual themselves more power in the mind-shaping process to dictate the norms in play.

## Conclusion

To briefly summarise, I have outlined the mind-shaping thesis and provided some an initially plausible cases of looping and shaping effects in psychiatry. I strengthened the case of mind-shaping processes in psychiatry by demonstrating the embedded non-epistemic values within clinical work and research; this showed that psychiatry is not primarily concerned with studying things in nature but has additional, social goals (e.g. treatment of disorder). In addition to this, I made the case that psychiatry constructs the roles of clinicians, researchers and patients along the lines of these non-epistemic values, and thus these roles are embedded in social norms much like the roles utilized in mind-shaping (such as the role of ‘teacher’). Psychiatry additionally makes use of disorder concepts in mind-shaping ways; they demarcate the relevant space for psychiatry to coordinate its scientific efforts, prescribe particular behaviour for those being categorised (which creates looping effects as individuals respond to this categorization), and they also help clinicians and researchers understand the person being categorized. All of these features make the case for psychiatry being implicated in mind-shaping processes, I claim.

With this comes a number of important consequences that researchers should consider, namely, the normative implications of concept application and construction, as well as the slipperiness of shifting disorder phenomena that concepts and categories are meant to capture. Researchers may prefer a pluralistic approach moving forward by generating many different models informed by a variety of lived experiences and patient needs. While I think this is a promising start, I have concerns that this does not necessitate critical engagement between models (see Alexandrova, 2018, and Russell, 2023 for discussion on pluralism and enactive psychiatry). Additional practices may need to be adopted to facilitate critical discussion among researchers, clinicians and patients alike, guided by feminist philosophy of science.

## Footnotes

1 The distinction between epistemic and non-epistemic values has been argued to be poorly defined and unhelpful, with some feminist philosophers of science rejecting the distinction (Longino 1996; Rooney 1992).

2 The picture I have presented here is a little one-sided in that it is psychiatry shaping individuals that would be patients, rather than the other way around. This raises a legitimate question as to why this is; why is psychiatry itself not being shaped by the activity of individuals with mental disorder? I do not deny that this is the case; there is good evidence to suggest that psychiatry is very responsive to the behaviour of patients and thus itself shaped by them. As I note in Sect. 3, there appears to be shaping effects to the DSM as a result of widening identification with diagnostic labels, and this generates a kind of looping effect where patients play an active role in the looping processes. Nevertheless, I would add that the shaping effects of psychiatry have more normative force; the norm-setting by psychiatric practice is more likely to have uptake due to “identity power” (Fricker 2007). That is, researchers and clinicians in psychiatry may have more power within the shaping process because their identity lends them such, and this may be as a result of the norms of these identities. Researchers, for example, have a lot more resources at their disposal to change the scope of knowledge and understanding; they not only have access to particular measurement tools (Cooper 2017) but also access to things like academic publishing, which we’ve already (normatively) legitimatised as a source of knowledge and truth. So we assume that what researchers say is true and well informed, which makes us want to ‘fall in line’ with what they say. This is not an anti-intellectualist point, only to say that the reason that researchers may hold such power is partially down to social and historical processes, as much as their genuine expertise.

3 Heyman et al. (2021) note positives to the TikTok Tourette’s phenomenon as individuals on the social media platform report experiences of support, recognition and belonging. The authors also note that the explanation of what functional tics are can help reduce the symptoms experienced. This implies to me that there is also great power in the understanding that concepts of mental disorder afford.

4 Conelea et al. (2022) advocate, in solution to this, not only caution and thoughtfulness in communication between researchers and the public but also a person-centred approach to research in general with a focus on “alleviating aspects of stigma and impairment that directly impact quality of life” (p. 6). This supports parts of my positive thesis below where I emphasise caution but also the necessity of discussion with patients about the prescriptions implicit in disorder concepts.
